# Centering Weight Management Clinical Decision Support in Primary Care on Patients With Obesity and Practitioners: A Proof‐Of‐Concept Study

**DOI:** 10.1002/osp4.70056

**Published:** 2025-02-12

**Authors:** Kimberly A. Gudzune, Jessica L. Schwartz, Kelly Olsson, Erik Almazan, Thomas Grader Beck, Jyotsna Ghosh, Wendy L. Bennett, Jeanne M. Clark

**Affiliations:** ^1^ Division of General Internal Medicine Johns Hopkins University School of Medicine Baltimore Maryland USA; ^2^ Welch Center for Prevention, Epidemiology, and Clinical Research Johns Hopkins Medical Institution Baltimore Maryland USA; ^3^ Division of Hospital Medicine Johns Hopkins University School of Medicine Baltimore Maryland USA; ^4^ Clinical Data Science and Evidence Novo Nordisk Inc. Plainsboro New Jersey USA; ^5^ Department of Medicine Brigham and Women's Hospital Boston Massachusetts USA; ^6^ Division of Rheumatology Johns Hopkins University School of Medicine Baltimore Maryland USA

**Keywords:** clinical decision support systems, obesity, primary health care

## Abstract

**Background:**

Clinical decision support systems (CDSS) are electronic health record tools that support practitioners' decision‐making at the point‐of‐care. CDSS may aid clinical care but are not often centered on patients or practitioners.

**Aims:**

To develop and preliminarily test a CDSS designed to support evidence‐based obesity treatment, promote a patient‐centered experience, and integrate with clinical workflows.

**Materials & Methods:**

The CDSS allowed patients to complete a pre‐visit questionnaire via the patient portal, which activated multiple elements for the primary care practitioner (PCP). A 3‐month proof‐of‐concept study was conducted among 10 PCPs at 5 clinics to determine usefulness, usability, and acceptability through validated surveys (mean score ≥ 2.5 signified positive outcome; max 5). Using t‐tests, pre‐post differences in PCPs' frequency of self‐reported clinical practices (1‐never; 5‐always) were examined.

**Results:**

Most PCPs were physicians with mean experience of 10.8 years (SD 7.5). Overall, mean scores for usefulness, usability, and acceptability were 3.2 (SD 0.8), 3.5 (SD 0.9), and 3.6 (SD 0.9), respectively. PCPs reported significant increases in three key clinical practices—counseling on behavioral interventions (3.1 vs. 3.9 [*p* < 0.01]), referring to weight‐loss programs (2.8 vs. 3.5 [*p* < 0.01]), and discussing anti‐obesity medications (3.3 vs. 3.8 [*p* = 0.02]).

**Conclusion:**

This weight management CDSS was useful and usable for PCPs and improved obesity‐related practice habits. Future studies need to evaluate its impact on patient outcomes.

## 1 Introduction

Interventions that effectively address obesity in primary care are critically needed. Counseling approaches, such as the 5As and motivational interviewing, may promote patients' behavior change in the primary care setting; however, few practitioners use these strategies appropriately in clinical practice [1, 2]. Prior studies have found that primary care practitioners (PCPs) lack time, training, and incentives to deliver intensive, comprehensive lifestyle interventions [3, 4]. However, research has found that patients want their PCP involved in their weight reduction plan [5]. PCPs desire simple strategies to incorporate obesity care into their practice [6, 7], which may be needed given their substantial gaps in knowledge and skills in obesity management [3, 4].

Since the passage of the Health Information Technology for Economic and Clinical Health Act in 2009 that incentivized electronic health record (EHR) use [8], a systematic review identified several studies that designed EHR tools to address obesity among adults in primary care settings [9]. Prior studies have found that individuals who received a diagnosis of obesity were more likely to report successful weight loss [10, 11], yet the rates of diagnosis were low. Therefore, many EHR tools have focused on increasing diagnoses of obesity [9]. For example, an observational study of EHR encounters found that patients with body mass index (BMI) ≥ 30 kg/m^2^ who received a diagnosis of obesity were significantly more likely to achieve a subsequent 5% weight loss within 15 months as compared to those individuals who did not receive this diagnosis [10]. Interestingly, this same study identified obesity treatment with anti‐obesity medication as the strongest predictor of achieving both 5% and 10% weight loss, despite only 3.3% of all adults with obesity in this sample receiving this treatment. This finding suggests that the diagnosis of obesity may prompt self‐directed weight loss attempts but does not necessarily increase the likelihood of medical treatment by a PCP.

Rather than focusing on diagnosis, clinical decision support systems (CDSS) are EHR tools that have the potential to support practitioners at the point‐of‐care to promote evidence‐based weight management guidelines [12]. Generally, CDSS have had positive impacts on process measures, such as increased ordering of preventive and treatment services and increased medical knowledge [13]. CDSS can support shared decision‐making in general, where practitioners and patients partner to reach treatment decisions [14]. A 2017 Cochrane systematic review of weight reduction interventions designed to change health professionals' behavior identified only one interventional study testing CDSS in an adult patient population [15]. Since this review, Fitzpatrick and colleagues published the results of a 6‐month cluster‐randomized trial comparing a best practice alert (BPA) for BMI ≥ 30 kg/m^2^ that prompted provision of a nutrition education handout (control clinics) versus referral to weight management programs (intervention clinics) [16]. While this BPA was associated with documenting an obesity diagnosis (31%), intervention clinics did not have significantly higher rates of weight management referrals than control clinics. Through in‐depth interviews with participating PCPs, the usability of the BPA was noted to be poor and PCPs felt that they needed additional education on the BPA and more information about the listed weight management programs [16]. Given their potential to increase the adoption of evidence‐based recommendations, more EHR interventions need to be designed with input from practitioners to improve the user experience [17].

In this study, an innovative CDSS was designed and tested in a proof‐of‐concept study with the main objective of estimating its usefulness, usability, acceptability, and use in the primary care setting. It was hypothesized that the CDSS would be useful for and usable in primary care settings and that it would be acceptable to and used by PCPs. Secondarily, changes pre‐post in PCPs' obesity care self‐efficacy and self‐reported clinical practice habits were examined.

## 2 Materials & Methods

### 2.1 CDSS Design

To inform the approach, a literature review to identify behavioral counseling interventions for obesity as well as EHR‐based tools for obesity in primary care settings was conducted [18]. The key lessons from the literature review were combined along with expert feedback from the research team, which included primary care and obesity medicine physicians, to design the CDSS in collaboration with the institutional EHR developer. Principles from both the 5As framework and motivational interviewing were incorporated into the CDSS, as these strategies have been associated with positive PCP and patient outcomes [1, 2]. The CDSS targeted adult patients with BMI ≥ 30 kg/m^2^. Other included features were clinical workflow integration, active alerts, and brevity for completion (< 5 min). As pre‐visit questionnaires may be useful to identify risk factors, patient‐entered pre‐visit questionnaires were incorporated into the CDSS. The CDSS identified potentially eligible patients and automatically sent them a pre‐visit questionnaire on weight management that they had the option to complete prior to their visit (no staff time was required). By collecting information before the visit, the CDSS synthesized patient‐entered data for PCPs with the goal of reducing the time spent during the encounter on collecting and interpreting information.

**FIGURE 1:**
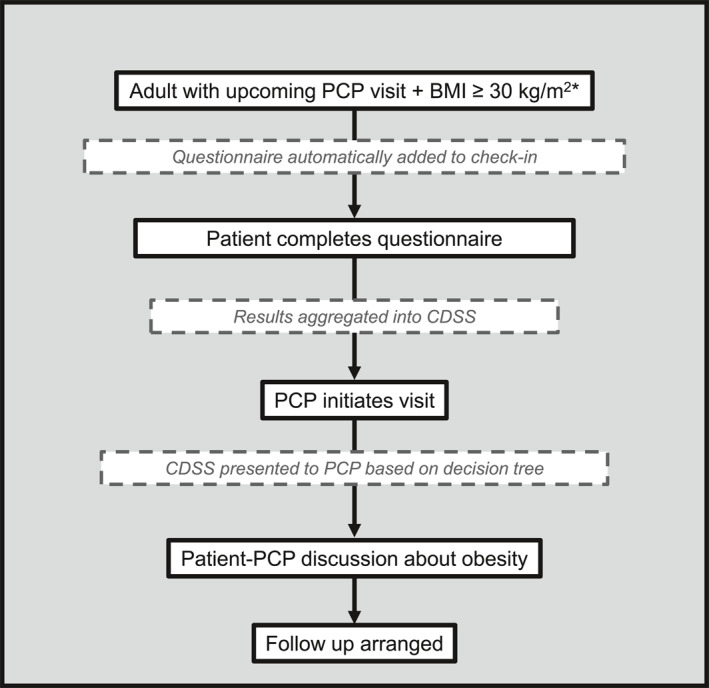
Overview of clinical decision support system workflow. This figure shows the workflow for the CDSS used in this study. Boxes with dashed lines indicate actions that the electronic health record performs as part of the CDSS. BMI, body mass index; CDSS, clinical decision support system; PCP, primary care practitioner. *Visits within 7 days scheduled with enrolled practitioner along with eligible BMI within the last 12 months in the electronic health record.

**FIGURE 2:**
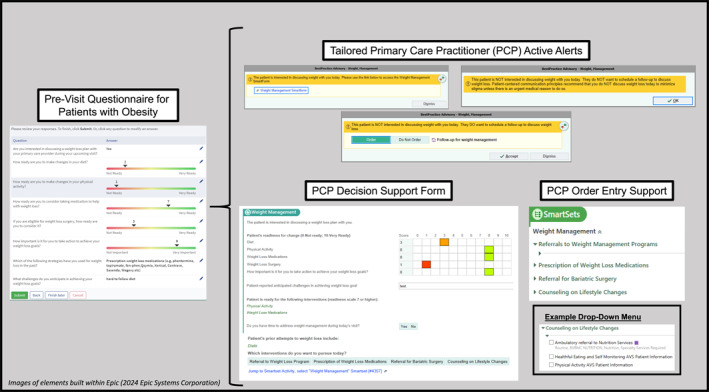
Key elements of weight management clinical decision support system for primary care settings. This figure contains images of the key elements of the CDSS used in this study: patient pre‐visit questionnaire, tailored active alerts for PCPs, PCP decision support form, and PCP order entry support. Figure does not provide an image of automated documentation for visit notes, although this element was a part of the CDSS. CDSS, clinical decision support system; PCP, primary care practitioner.

Overall, the CDSS aimed to address the following key objectives: 1. Promote a patient‐centered experience to avoid weight stigmatization; 2. Support evidence‐based obesity treatment among PCPs; 3. Facilitate PCPs' use of evidence‐based counseling approaches; 4. Integrate efficiently with PCPs' current workflows for decision‐support and documentation; and 5. Encourage use (and continued use) among PCPs over time. The CDSS design relied upon inputs from both patients and PCPs to create an approach centered on both patients and practitioners. Figure 1 provides an overview of the resulting CDSS workflow. The CDSS approach used multiple EHR elements , including automation to identify eligible patients; patient‐entered pre‐visit questionnaire that employed patient‐centered principles and motivational interviewing tools; BPAs tailored for specific patient scenarios; PCP decision‐support form (“SmartForm”); PCP order‐entry support (“SmartSet”) including pre‐populated orders and patient education materials; and automated documentation for visit notes (“SmartLink”). Figure 2 displays images of key CDSS elements, and Supporting Information S1: 1 provides additional details on the CDSS.

### 2.2 Proof‐Of‐Concept Study Design

A 3‐month single‐arm proof‐of‐concept study with the CDSS described above was conducted (December 2022‐May 2023). The primary outcomes were usefulness, usability, acceptability, intention to use, and use of the CDSS. As secondary objectives, PCPs' obesity care self‐efficacy and self‐reported clinical practice habits were compared pre‐post. The Johns Hopkins Institutional Review Board approved this study (IRB00316041).

### 2.3 Recruitment and Eligibility Criteria

Study participants were PCPs that were recruited from 5 primary care clinics from a large system‐owned multi‐specialty healthcare organization in Maryland. Clinic medical directors notified PCPs about the study via email and the research team also presented the study to PCPs at meetings. Interested PCPs were screened for eligibility by the research team, and eligible PCPs wanting to participate signed informed consent. The research team aimed to recruit at least 10 PCPs.

To be eligible, participants had to be at least 18 years of age; work as a PCP (i.e., physician, nurse practitioner, or physician assistant) at one of the five included primary care clinics; typically spend at least two half‐day sessions (8 h per week) delivering outpatient primary care services predominantly to adult patients (at least 50% of care delivered to adults (age ≥ 18 years)); and have at least four half‐day clinic sessions planned during specific 2‐week blocks for chart extraction (see below).

### 2.4 Intervention

The intervention consisted of two components—an orientation session and access to the CDSS. Each PCP participated in a one‐on‐one orientation session delivered by an obesity medicine physician where 1) the evidence‐based guidelines on adult weight management were reviewed [19, 20, 21], 2) weight bias in healthcare settings was discussed [22], and 3) the CDSS was introduced. Each orientation session lasted approximately 45 min. After this session, the CDSS was activated within the PCPs' profile in the EHR. The CDSS pre‐visit patient questionnaire only triggered for adult patients scheduled for routine follow‐up visits who had measured BMI ≥ 30 kg/m^2^ within the prior 12 months (as recorded within the EHR). Clinics provided a list of visit types used in their practice for routine follow‐up care as this differed by site (e.g., office visit, annual physical). The CDSS was not linked to new patient visits, urgent visits, or hospital discharge visits as weight management discussions may be inappropriate in these scenarios. Use of the CDSS practitioner elements was at the discretion of the PCP.

### 2.5 Data Collection

Two types of data were collected—survey data and chart extraction data. Participating PCPs completed surveys at baseline and 3 months. Using a standardized extraction form, the research team manually extracted information from the medical records of encounters with adult patients with BMI ≥ 30 kg/m^2^ on their visit date seen by included PCPs within 2‐week blocks at 3 time points—baseline, 2 weeks after CDSS activation, and 3 months after CDSS activation. At each of these time points, information was extracted from eligible medical records from 4 half‐day sessions during the allotted 2‐week timeframe (up to 4 weeks was considered for instances of vacation, PCP illness, etc.). Information was extracted from 128 records at baseline, 139 records at 2 weeks, and 115 records at 3 months.

### 2.6 Measures

The primary outcomes were PCP‐rated usefulness, usability, acceptability, and intention to use the CDSS assessed via questionnaires at 3 months. CDSS use was determined via manual chart extraction data at 2 weeks and 3 months. *Usefulness* and *usability* were determined using adapted versions of the Perceived Usefulness and Perceived Ease of Use surveys from the Technology Acceptance Model (TAM), respectively [23, 24, 25, 26]. Summary scores for each of these elements were created, and individual item scores were also assessed. Of note, prior research using the TAM scales within the context of obesity‐related EHR tools has interpreted mean item scores of 2.5 as “neutral” [25], and this same threshold was used to indicate usefulness and usability in this study. *Acceptability*, including appropriateness for the primary care setting, was determined using adapted measures from Weiner and colleagues [27]. A summary score for acceptability was created and individual item scores were also assessed. *Intention to use* was assessed using a modified scale from Constantinides and colleagues [28]. Chart extraction data were used to capture actual *CDSS use* among all eligible encounters with adult patients with obesity at 2 weeks (initial adoption) and 3 months (persistence of use). For the patient elements, the proportion of visits where the pre‐visit questionnaire was sent to an adult patient with obesity was extracted as well as the proportion of visits where this questionnaire was completed by the patient. The research team defined PCP CDSS use if any PCP element was used during that visit, since different elements may be appropriate based on varying clinical contexts for each patient (i.e., BPA acknowledged, SmartForm used, SmartSet used, or SmartLink used). The proportion of visits where CDSS was used by the PCP was calculated.

The secondary outcomes were PCPs' obesity care self‐efficacy and self‐reported clinical practice habits. Survey data were used to determine obesity care self‐efficacy and practice habits from a previously validated measure at baseline and 3 months [29]. Mean scores pre‐post for each item on these measures were compared.

At baseline, demographics, prior EHR and weight management training, perceived EHR use, and attitudes toward EHR use were captured. Attitudes toward individuals with obesity [30] were assessed. The frequency of documented weight management care elements (i.e., obesity diagnosis, weight management plan, counseling approach, and weight‐related diagnosis code) at baseline, 2 weeks, and 3 months was also explored and captured through chart extraction.

### 2.7 Statistical Analysis

For the primary outcomes of perceived usefulness, usability, acceptability, and intention to use, mean values for each questionnaire item were calculated and percent who agreed with each item was reported. For use, the number and percentage of records where CDSS elements were used at 2 weeks and 3 months was reported.

For the secondary outcomes of self‐efficacy and self‐reported clinical practice habits, pre‐post mean differences for each questionnaire item and examined with paired t‐tests were calculated. Pre‐post differences of these secondary outcomes were examined using the non‐parametric Wilcoxon signed rank test, as some outcomes did not meet the normality assumption. Given that results were overall similar whether pre‐post differences were examined with parametric or non‐parametric tests, only the mean and *t*‐test results were reported. Results using the non‐parametric tests are available upon request.

## 3 Results

Overall, 10 PCPs were recruited to participate from 5 different clinics. Table 1 shows PCPs' baseline characteristics. Most practitioner participants were physicians with mean primary care experience of 10.8 years (SD 7.5). On average, they used the EHR for 7.3 years (SD 2.4) and 70% agreed that CDSS alerts help them make decisions. They reported low quality of prior obesity education received—none rated their prior obesity training as very good/excellent in any setting (i.e., graduate medical education, post‐graduate training, continuing medical education). PCPs strongly agreed that obesity is a chronic disease associated with serious medical conditions and that they made accommodations for patients with obesity in their clinics (e.g., large blood pressure cuffs, large examination gowns, armless chairs). PCPs disagreed that patients with obesity inspired a lack of empathy or negative reactions; they disagreed that scare tactics were an appropriate motivational tool.

**TABLE 1:** Baseline characteristics of primary care practitioners.

	PCPs (*n* = 10)	
*Demographics*		
Years of primary care experience, mean (SD)	10.8 (7.5)	
PCP type, %		
Physician	70%	
Nurse practitioner	30%	
Physician assistant	0	
Clinical care focused^a^, %	70%	
Women, %	80%	
Race, %		
White	80%	
Black	0	
Asian	20%	

*Experience and attitudes: EHR*	Mean score (SD)	% Agree/Strongly Agree
Years of experience using EHR	7.3 (2.4)	—
Quality of training using EHR, mean score (SD)	3.8 (1.1)	—
Frequency of information use from CDSS alerts, mean score (SD)	3.4 (0.8)	—
Attitudes toward CDSS alerts during patient encounter		
Help me make decisions	3.8 (0.6)	70%
Are very disruptive to my workflow	2.7 (0.9)	30%
I often close CDSS alerts without reading prompts	3.2 (0.8)	40%
I really dislike CDSS alerts and find them annoying	2.9 (1.0)	20%

*Experience and attitudes: Obesity*	Mean score (SD)	% Agree/Strongly Agree
Quality of weight management training^b^		
Graduate medical training	1.9 (0.6)	—
Post‐graduate training (e.g., residency)	2.0 (0.9)	—
Continuing medical education	2.2 (0.7)	—
Beliefs and attitudes about obesity and patients with obesity^c^		
Obesity is a chronic disease	4.5 (0.5)	100%
Obesity is associated with serious medical conditions	4.6 (0.5)	100%
Most patients with obesity are well aware of the health risks of obesity	3.1 (1.1)	40%
It is difficult for me to feel empathy for a patient with obesity	1.6 (0.5)	0%
I have negative reactions toward the appearance of patients with obesity	1.8 (0.8)	0%
I feel uncomfortable when examining a patient with obesity	1.8 (0.6)	0%
I make accommodations for patients with obesity	4.3 (0.5)	100%
Most patients with obesity could reach a normal BMI if they were motivated to do so	2.4 (0.8)	10%
It is acceptable to use “scare tactics” to motivate patients with obesity to lose weight	1.6 (0.7)	0%
Most patients with obesity will not lose a significant amount of weight	2.9 (0.6)	10%
For most patients with obesity, long‐term maintenance of weight loss is impossible	2.2 (0.6)	0%

*Note:* All scores assessed on five‐point Likert scales—quality of training: poor (1), fair (2), good (3), very good (4), excellent (5); frequency of use: never (1), rarely (2), sometimes (3), usually (4), always (5); agreement with belief/attitude statements: strongly disagree (1), disagree (2), neutral (3), agree (4), strongly agree (5).

Abbreviations: CDSS, clinical decision support system; EHR, electronic health record; PCP, primary care practitioner.

^a^ Clinical care focused defined as having five or more half‐day sessions per week in primary care.

^b^ Quality of trainings rated as excellent (5), very good (4), good (3), fair (2), poor (1) and did not receive (0).

^c^ Questionnaire modified from Foster and colleagues [28].

### 3.1 Primary Outcomes

Table 2 shows the outcomes of perceived usefulness, usability, intention to use, and acceptability at 3 months. The mean score on the TAM usefulness scale was 3.2 (SD 0.8), and all individual items composing the usefulness scale had mean scores greater than 2.5, suggesting that PCPs found the CDSS useful. Individual usefulness item score was particularly high in recommending the tool to a colleague to help with weight management; however, others were low—for example, few PCPs agreed that the CDSS increased productivity. The mean score on the TAM usability scale was 3.5 (SD 0.9)—PCPs found the CDSS clear and easy‐to‐use. PCPs rated the degree to which each intervention component impacted their delivery of obesity treatment during visits, and ratings of “a lot” or “a great deal” were considered positive impacts. All PCPs (100%) positively rated the patient questionnaire, followed by the decision‐support form (80%), orientation session (70%), order‐entry support (50%), and automated documentation (0%). Overall, 60% of PCPs indicated that it was probable that they would continue to use the CDSS. Regarding acceptability for weight management counseling in the primary care setting, the overall acceptability score was 3.6 (SD 0.9). Domains related to appropriateness of the CDSS for weight management in the primary care setting were highly rated, although few PCPs reported have an extraordinary confidence in the CDSS in supporting their counseling.

**TABLE 2:** Primary care practitioners' (*n* = 10) usefulness, usability, acceptability, and intention to use the clinical decision support system at 3 months.

	Mean score (SD)	% Agree/Strongly Agree
Usefulness^a^		
I would recommend the tool to a colleague who wants to help their patients lose weight	3.9 (1.0)	70%
I found the tool useful in my job	3.5 (1.0)	60%
Using the tool made it easier to do my job	3.2 (0.9)	50%
Using the tool improved my job performance	3.0 (0.8)	30%
Using the tool helped me accomplish tasks more quickly	3.0 (0.8)	30%
Using the tool enhanced my effectiveness on the job	3.0 (0.8)	30%
Using the tool increased my productivity	2.9 (0.9)	30%
*Overall mean usefulness score*	3.2 (0.8)	—
Usability^a^		
My interaction with the tool was clear and understandable	3.7 (0.9)	80%
Learning to use the tool was easy for me	3.6 (1.1)	60%
It was easy to become skillful using the tool	3.5 (1.0)	60%
I found the tool easy to use	3.4 (0.8)	60%
I found the tool flexible to interact with	3.3 (0.8)	50%
I found it easy to get the tool to do what I wanted	3.2 (1.0)	40%
*Overall mean usability score*	3.5 (0.9)	—
Acceptability for weight management counseling in primary care^b^		
The tool seems suitable as a support for this counseling in primary care	4.1 (1.0)	80%
The tool seems applicable as a support for this counseling in primary care	4.1 (1.0)	80%
The tool seems fitting as a support for this counseling in primary care	3.9 (1.2)	70%
The tool seems like a good match for primary care setting	3.8 (1.2)	60%
I welcome the tool as a support for this counseling	3.7 (1.2)	60%
The tool is appealing to me as a support for this counseling	3.5 (1.1)	50%
The tool meets my approval as a support for this counseling	3.4 (0.8)	60%
I like the tool as a support for this counseling	3.4 (1.1)	40%
I am perfectly satisfied with the tool as a support for this counseling	2.9 (1.0)	40%
I have an extraordinary amount of confidence in the tool as a support for this counseling	2.9 (0.7)	20%
*Overall mean acceptability score*	3.6 (0.9)	—
Intention to use^c^		
It is probable that I will continue using the tool	3.6 (0.8)	60%
I intend to continue to use the tool	3.4 (1.0)	50%

*Note:* All outcomes assessed on 5‐point Likert scales—strongly disagree (1), disagree (2), neutral (3), agree (4), strongly agree (5).

^a^ Usefulness and usability assessed via adapted versions of the Technology Acceptance Model Perceived Usefulness and Perceived Ease of Use Surveys [21, 22, 23, 24].

^b^ Acceptability and appropriateness assessed via Weiner and colleagues' measure [25].

^c^ Intention to use assessed via adapted version of Constantinides and colleagues' scale [26].

CDSS use was examined among encounters with adult patients with obesity at 2 weeks and 3 months (Table 3). At both time points, a substantial number of patients were not sent the pre‐visit questionnaire (40%–52%). During chart extraction, this occurred for several reasons: 1) patient did not have a BMI measured within the last 12 months; 2) patient's prior BMI was < 30 kg/m^2^; or 3) patient was scheduled for a certain visit type, such as annual checkup, that was not included in the algorithm to trigger a pre‐visit questionnaire to be sent. Among patients who were sent the pre‐visit questionnaire, only 21.4% at 2 weeks and 37.5% at 3 months completed it. Across the entire study duration, the BPA fired 219 times—126 patients (57.5%) reported interest in discussing weight loss at the visit, 6 (2.7%) were interested in scheduling a future visit to discuss weight loss, and 87 (39.7%) did not want to discuss the topic during the current visit or schedule a future visit to discuss weight loss. When the patient questionnaire was completed, PCPs used the CDSS in over 70% of encounters at both time points (per chart extraction).

**TABLE 3:** Use of the clinical decision support system during the study period.

	Initial adoption (2 weeks)	Persistent use (3 months)
# PCPs with available records	10	9^a^
# Extracted encounters among adults with obesity	138	115
Use, *n* (%)		
Pre‐visit questionnaire sent to patient	83 (60.1%)	56 (48.7%)
Patient questionnaire completed	18 (13.0%)	21 (18.3%)
Questionnaire completed if patient questionnaire sent^b^	18 (21.7%)	21 (37.5%)
CDSS used by PCP	23 (16.7%)	19 (16.5%)
CDSS used by PCP if patient questionnaire completed^c^	16 (88.9%)	15 (71.4%)

Abbreviations: CDSS, clinical decision support system; PCP, primary care practitioner.

^a^ 1 PCP was unable to contribute 3‐month chart data due to unexpected medical leave, and therefore, had no notes within the allowed timeframe within the study protocol.

^b^ Denominator for this row is patients who were sent the questionnaire through the electronic medical record system and thus had the opportunity to complete the questionnaire (*n* = 83 and *n* = 56 at 2 weeks and 3 months, respectively) rather than all encounters extracted (*n* = 138 and *n* = 115 at 2 weeks and 3 months, respectively).

^c^ Denominator for this row is patients who completed the questionnaire (*n* = 18 and *n* = 21 at 2 weeks and 3 months, respectively) rather than all encounters extracted (*n* = 138 and *n* = 115 at 2 weeks and 3 months, respectively).

### 3.2 Secondary Outcomes

Table 4 shows the scores for self‐efficacy and self‐reported clinical practice habits at baseline and 3 months. Overall, PCPs had statistically significant increases in obesity care self‐efficacy in almost every domain. There were statistically significant increases in certain obesity care practice habits—counseling on behavioral interventions, referring to weight‐loss programs, and discussing anti‐obesity medications. Supporting Information S1: 2 and 3 display changes in self‐efficacy and self‐reported practice habits, which show the distribution of categorical responses for each item at baseline and 3 months. At 3 months, most PCPs reported that they were fairly or very confident in all obesity care self‐efficacy domains, and the majority of PCPs reported that they often or always delivered nearly all obesity care clinical practice habits. Supporting Information S1: 4 visualizes individual‐level changes in overall mean self‐efficacy and clinical practice habits at each time point. All PCPs had an increase in overall mean self‐efficacy score over time.

**TABLE 4:** Primary care practitioners' (*n* = 10) obesity treatment self‐efficacy and self‐reported obesity care clinical practice habits, pre and post.

	Mean score (SD)		*p*‐value^a^
	Baseline	3‐month	
Self‐efficacy^b^			
Assessing the degree of overweight or obesity	3.5 (1.1)	4.3 (1.0)	< 0.01
Advising patients about the potential health benefits of weight loss	3.9 (0.6)	4.6 (0.5)	< 0.01
Counseling patients on behavioral interventions for weight loss	3.2 (0.8)	3.9 (0.7)	0.01
Counseling patients on dietary changes for weight loss	3.6 (0.5)	3.9 (0.7)	0.30
Counseling patients on physical activity for weight loss	3.3 (0.7)	3.9 (0.9)	0.08
Referring patients with obesity to a weight‐loss program	3.4 (1.2)	4.6 (0.5)	< 0.01
Responding to patient's questions regarding lifestyle changes	3.5 (0.7)	4.4 (0.5)	< 0.01
Discussing anti‐obesity medications	3.1 (1.1)	4.6 (0.5)	< 0.01
Self‐reported clinical practice habits^b^			
Assess the degree of overweight or obesity	3.6 (1.0)	4.1 (0.7)	0.10
Advise patients about the potential health benefits of weight loss	3.6 (0.5)	3.7 (0.5)	0.59
Counsel patients on behavioral interventions for weight loss	3.1 (0.7)	3.9 (0.6)	< 0.01
Counsel patients on dietary changes for weight loss	3.7 (0.5)	3.8 (0.4)	0.34
Counsel patients on physical activity for weight loss	3.5 (0.5)	3.7 (0.5)	0.34
Refer patients with obesity to a weight‐loss program	2.8 (0.8)	3.5 (0.7)	< 0.01
Discuss anti‐obesity medications	3.3 (0.7)	3.8 (0.8)	0.02

*Note:* All scores assessed on five‐point Likert scales—confidence: not at all (1), slightly (2), somewhat (3), fairly (4), very confident (5); frequency of practice habits: never (1), rarely (2), sometimes (3), usually (4), always (5); agreement with belief/attitude statements: strongly disagree (1), disagree (2), neutral (3), agree (4), strongly agree (5).

^a^ *p*‐values calculated using t‐tests.

^b^ Questionnaire from Wilder and colleagues [27].

The frequency of documented weight management care elements identified through chart extraction was also explored; however, variability in PCP documentation practices limited confidence in these results. Supporting Information S1: 5 displays these results and provides additional details on the challenges and limitations regarding these measures.

## 4 Discussion

This proof‐of‐concept study was conducted with 10 PCPs in real‐world clinical practice settings examining a CDSS to support evidence‐based obesity treatment among adults. The CDSS was rated as having overall usefulness. PCPs found the CDSS clear, easy‐to‐use, and appropriate for obesity management in the primary care setting. PCPs also had statistically significant increases in obesity care self‐efficacy in nearly every domain assessed. Three obesity care practice habits also had statistically significant increases, including counseling on behavioral interventions, referring to weight‐loss programs, and discussing anti‐obesity medications.

Prior studies of EHR tools designed to support weight management in primary care have primarily focused on engaging PCPs [9, 16, 31, 32]. In contrast, the CDSS in this study engaged both patients and PCPs. First, patients completed a questionnaire where they could indicate their interest in discussing obesity treatment with their PCP, which then triggered a BPA to be presented to the PCP that was linked to a decision‐support form to assist with counseling and treatment selection as well as to an order set that included referrals, medications and patient educational handouts. This study found that if a patient completed the questionnaire, PCP use of the CDSS was high—this finding differs from the low PCP use found for EHR tools that only engaged PCPs [16, 31, 32]. PCPs may not always feel confident about how to sensitively approach the topic of weight management with their patients [4]. By having the CDSS first engage patients to assess their interest in discussing weight management, it is suspected that PCPs' concerns about raising the topic of obesity may have reduced and thus resulted in high CDSS use when the patient questionnaire was completed. In fact, all PCPs rated the patient questionnaire positively when evaluating all components of the CDSS. Communication between patients and practitioners has been identified as a barrier to obesity treatment [33], and strategies that support shared decision‐making, such as CDSS [14], may improve treatment.

While PCPs in this study positively rated the usefulness, usability, and acceptability of the CDSS for weight management counseling in the primary care setting, these ratings may be viewed as moderate rather than high magnitude (overall mean scores of 3.2, 3.5 and 3.6, respectively, where maximum value possible was 5). In addition, only 50% of PCPs in this study intended to continue using the CDSS. A few factors may be contributing to these results. PCPs may negatively view EHR tools in general, as they give EHRs poor usability scores and associate EHRs with burnout [34]. In fact, one study found that PCPs spend nearly 6 h each workday interacting with the EHR—and this time occurred both during and after clinic hours [35]. PCPs may lack time during routine visits to address obesity, as research has found that they do not have enough time to provide all guideline‐recommended primary care services [36]. In addition, there are well‐documented gaps in obesity education during medical training [37], which may contribute to PCPs being unprepared to manage obesity. Given these challenges, a CDSS alone may be insufficient.

Instead, the primary care workflow may need to be modified to address obesity. For example, a large randomized clinical trial is currently ongoing which is combining weight management dedicated visits in primary care (workflow modification) with a CDSS that engages both patients and PCPs [38, 39]. A recent pilot study developed a CDSS tool that supports PCP referral of patients to an obesity medicine physician rather than adding obesity treatment to the PCPs' scope of practice [40], which showed positive weight loss outcomes among referred patients. PCPs may alternatively desire referral to behavioral weight management teams, which also has demonstrated efficacy for weight reduction and weight loss maintenance [41, 42]. Despite the moderate ratings of the CDSS in this study, PCPs reported significant improvements in self‐efficacy across multiple obesity care domains as well as self‐reported clinical practice habits within domains of behavior change counseling, referral to weight‐loss programs, and discussing anti‐obesity medications. Given these promising findings, further refinements of the intervention, such as combining the CDSS with workflow modifications, should be considered prior to additional testing. Such changes may improve PCP efficiency, productivity, and satisfaction with the intervention, which were relatively low in this study. Future research is needed to determine the impact of this tool on patient outcomes.

This proof‐of‐concept study informed the following possible future modifications to this CDSS. The pre‐visit questionnaire was not sent to all eligible patients, which occurred when: 1) the patient did not have a BMI measured within the last 12 months; 2) the patient's prior BMI was less than 30 kg/m^2^; or 3) the patient was scheduled with certain visit types not linked to the questionnaire. Patients who did not have active accounts within the patient portal would also be unable to receive the pre‐visit questionnaire. In future research, it may be appropriate to expand the eligibility criteria for this questionnaire to allow use with additional visit types and among adults with a prior BMI ≥ 25 kg/m^2^ as this strategy would capture more patients who might potentially benefit from treatment. Clinics may also consider using tablets to increase the completion of questionnaires at the point‐of‐care, which may be particularly useful if the CDSS is combined with dedicated weight management visits [38, 39]. Among eligible patients who were sent the pre‐visit questionnaire in this study, less than half completed it—37.5% of patients at 3 months. Prior research has found variable rates of pre‐visit completion of EHR questionnaires [43, 44, 45], and achieving high patient completion rates may require flexibility in administration, including via EHR, paper, and phone [44]. Currently, there is no model for patient‐reported questionnaires that has been demonstrated to work in all contexts [46]; therefore, stakeholder participation in the design is likely the key to future success. Implementing clinic‐based strategies to encourage and increase pre‐visit questionnaire completion may be key (e.g., tablets in waiting room, medical assistants aid patients during rooming process). Given that PCPs rated the pre‐visit questionnaire highly and used the CDSS frequently when the patient completed the assessment, additional efforts to increase patient completion might potentially increase PCPs' delivery of evidence‐based obesity treatment.

This study has several limitations. First, this study was small, single‐arm, and occurred within a single health system—this design is appropriate for rapid evaluations of interventions but limits the generalizability of the findings. PCPs who were willing to participate were recruited in this study, who may be more interested in obesity treatment than other PCPs as suggested by their generally positive attitudes and approach toward patients with obesity (e.g., making accommodations, viewing obesity as a chronic disease). The small sample size prevented comparison of outcomes among primary care physicians, nurse practitioners and physician assistants. Future studies should consider recruiting a sample that may enable comparisons between these PCP types. In addition, the health system where this study occurred began an initiative to increase obesity diagnosis and documentation during the study period, which may have impacted the results. This study focused on determining outcomes among PCPs rather than examining patient outcomes. While usability and acceptability of the CDSS to PCPs is essential in considering an EHR‐based tool to treat obesity, future research needs to examine patient outcomes and increase patient engagement with the pre‐visit questionnaire. The research team did not have access to data on EHR orders placed (e.g., prescribing medication, placing referrals) for this pilot study. Inclusion of such data in future studies may better understand the impact of a CDSS on PCPs' behaviors.

In summary, a CDSS was created that provided evidence‐based obesity management decision‐support and patient‐centered care, which PCPs found useful and usable. The intervention was also associated with increased obesity care self‐efficacy. Future studies are needed to test a modified tool that addresses the aforementioned limitations and assesses patient outcomes.

## Conflicts of Interest

This research was funded by Novo Nordisk. KAG served as the medical director for the American Board of Obesity Medicine, has been a paid scientific advisor to Novo Nordisk and Eli Lilly, receives royalties from the Johns Hopkins ACG System, and received honoraria from PRI‐MED and the American College of Cardiology for educational events. Since completing this work, KAG joined the American Board of Obesity Medicine Foundation as an employee. KO is an employee of Novo Nordisk. JMC has been a paid scientific advisor to Boehringer Ingelheim and received writing support from Novo Nordisk for a manuscript on nonalcoholic fatty liver disease. Since completing this work, JMC joined the faculty of the Rutgers Robert Wood Johnson Medical School. All other authors declare that they have no competing interests.

## Supporting information

Supporting Information S1
