# Supplementary material for: Centering Weight Management Clinical Decision Support in Primary Care on Patients With Obesity and Practitioners: A Proof‐Of‐Concept Study
Source: Obes Sci Pract. 2025 Feb 12;11(1):e70056. doi: 10.1002/osp4.70056 (PMC11815222; doi:10.1002/osp4.70056)
Supplement: Supplementary file 1 — Supporting Information S1 [file OSP4-11-e70056-s001.docx]

**Supplemental Materials for “*Centering obesity clinical decision support in primary care on patients with obesity and practitioners: A proof-of-concept study*”**

**Table of Contents**

|  |  | **Page** |
| --- | --- | --- |
| Supplemental Materials 1. | Detailed Description of Clinical Decision Support System | 4 |
| Supplemental Materials 2. | Self-Efficacy Ratings at Baseline and 3 Months | 13 |
| Supplemental Materials 3. | Frequency of Self-Reported Clinical Practice Habits at Baseline and 3 Months | 14 |
| Supplemental Materials 4. | Overall Mean Self-Efficacy and Self-Reported Clinical Practice Habits at Baseline and 3 Months, by Individual Primary Care Practitioner | 15 |
| Supplemental Materials 5. | Chart Extraction Data: Results and Limitations | 16 |
|  |  |  |

**Supplemental Materials 1. Detailed Description of Clinical Decision Support System**

**Overall CDSS Design.** The CDSS aimed to address the following 5 key objectives:

- Promote a patient-centered experience to avoid weight stigmatization
- Support evidence-based obesity treatment among PCPs
- Facilitate PCPs’ use of evidence-based counseling approaches
- Integrate efficiently with PCPs’ current workflows for decision-support and documentation
- Encourage use (and continued use) among PCPs over time

Therefore, the CDSS design relied upon inputs from both patients and the PCPs to create an approach centered on both patients and practitioners. The resulting CDSS approach used multiple electronic health record (EHR) elements including:

- Automation to identify eligible patients
- Patient-entered pre-visit questionnaire
- Best practice alerts
- PCP decision-support form (“SmartForm”)
- PCP order-entry support (“SmartSet”) including patient education materials
- Automated documentation for visit notes (“SmartLink”)

In the text that follows, screenshots of the various CDSS features that were built within the Epic EHR (2024 Epic Systems Corporation) are provided. MyChart® is a registered trademark of Epic Systems Corporation.

**Patient Eligibility.** The tool was set to identify adult patients (age ≥ 18 years) who were scheduled to have an outpatient visit within the next 7 days with an enrolled PCP. If the patient’s most recent body mass index (BMI) in the EHR was ≥ 30 kg/m^2^ (class I obesity or greater), then the weight management questionnaire was automatically added to group of questionnaires for the patient to complete at check-in – either e-check-in through the online patient portal or check-in at in-person kiosks. Of note, the BMI value had to be measured within the last 12 months to trigger release of the questionnaire. The check-in system encourages, but does not require, patients to complete all questionnaires. Patients who do not use the patient portal or kiosk to check-in may not have the opportunity to complete questionnaires. Primary care visits can be specified into a variety of visit types. Each clinic provided the visit types that they typically used for routine follow-up to route pre-visit questionnaires to appropriate patients. The questionnaire was prevented from being added to the patient’s queue for certain visit types (e.g., urgent visit, hospital discharge visit), as it could be inappropriate to discuss weight management in these scenarios.

**Patient Questionnaire.** A patient-centered approach was applied when designing the questionnaire with the aim of reducing weight stigmatization. Therefore, patients were first presented with the following single question:


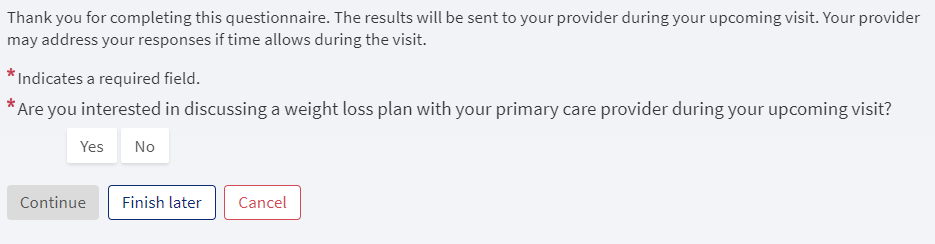


If the patient indicated that they did not want to discuss weight loss with their PCP, a second question appeared asking if they were interested in discussing weight loss at follow-up visit:


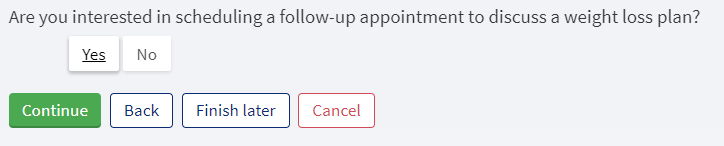


This strategy aimed to allow patients to indicate that discussing weight loss was not appropriate today, and alert to their openness to discussing weight loss in the future. After responding to this second question, the questionnaire was complete for these patients. However, if the patient indicated that they did want to discuss weight loss with their PCP today, then a different screen was presented that included options for self-assessment of readiness-for-change, obesity-related history, past weight-loss strategies, and current challenges in weight-loss goals. Readiness and importance rulers are tools used in motivational interviewing, which was used in the tool. The following is an example of one of the rulers:


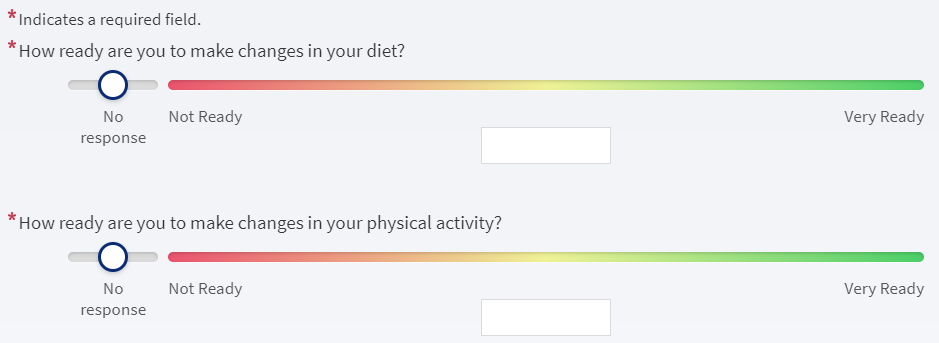


Readiness and importance rulers were used to gather data on key obesity treatments. Information on previous weight-loss strategies was collected via a list of pre-populated options, and current challenges to achieving weight-loss goals was reported via free text. To ensure a patient-centered experience, only patients who indicated that they wanted to discuss weight loss with their PCP today were presented these options. Prior to submitting the questionnaire, patients were able to review their answers:


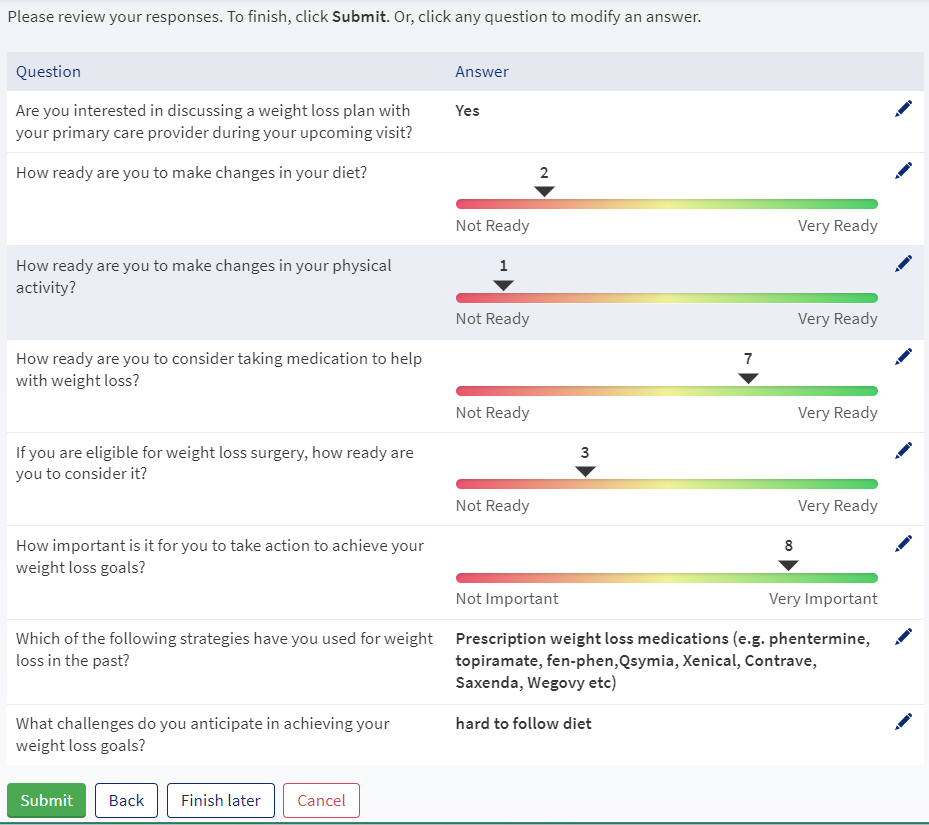


**Best Practice Alert (BPA).** BPAs are active alerts that pop-up when PCPs open a patient encounter, and require PCPs to click to acknowledge a message, statement or alert. The tool aggregated the results from the patient’s self-assessment entries to present the PCP with a tailored BPA. The BPA was designed to pop up when two conditions were met: 1) PCP opened the patient’s chart for the visit; and 2) appointment status was “arrived.” This strategy was put in place so that the BPA displayed when the PCP was seeing the patient, rather than during pre-charting (i.e., preparatory review before the patient arrives for their appointment). “Arrived” status was available for both in-person and video visits. Of note, if the patient did not complete the questionnaire, no BPA was displayed. Based on the patient questionnaire responses, the PCP received one of three BPAs:

Scenario 1: Patient does NOT want to address weight management now or at a follow-up appointment


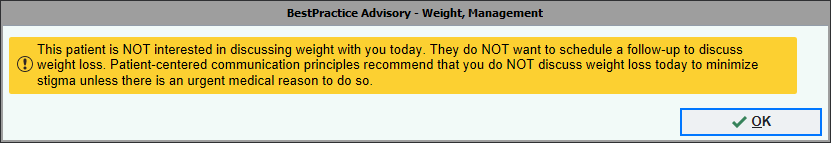


In scenario 1, the BPA aimed to support PCPs in creating a patient-centered approach by reinforcing that weight should not be discussed at this visit. In addition, the CDSS was structured so that these patients would not be prompted to complete questionnaire again for 12 months.

Scenario 2: Patient does NOT want to address weight management today, but does want to discuss it at a follow-up appointment


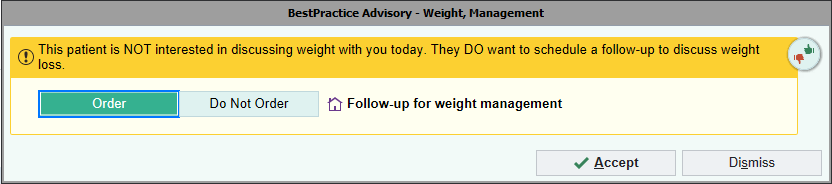


In scenario 2, the BPA again aims to encourage PCPs to avoid discussing weight loss at this visit, but also provides the ability to order a specific follow-up visit for this patient to discuss weight management from the BPA. The follow-up visit order was pre-populated with “weight management” as the visit reason and within the timeframe of 3 months to reduce PCP burden. These patients will receive the weight management questionnaire again at their next visit. In both scenarios 1 and 2, the PCP was restricted from accessing the weight management decision-support form manually by having an alert statement be given if this was attempted. This strategy was put in place to reinforce to the PCP that this patient does not want to discuss weight loss.

Scenario 3: Patient DOES want to address weight management today


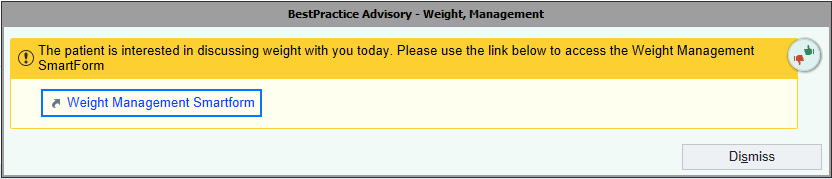


In scenario 3, the PCP could launch the decision-support form (“SmartForm”) from the BPA. The CDSS was designed so that PCPs could also access the Weight Management SmartForm through other routes, in the event that they dismissed the BPA at pop-up. Of note, patients continued to receive the weight management questionnaire at upcoming visit check-ins every 3 months to allow for tracking change over time.

**Weight Management SmartForm.** The Weight Management SmartForm displayed the patient’s responses to the initial questionnaire. As stated above, PCPs accessing this form for patients who did not want to discuss weight loss on the day of the visit (scenarios 1 or 2) would see a statement reinforcing this request. When the patient did want to discuss weight loss (scenario 3), the SmartForm displayed their responses to the PCP. **Supplemental Figure 2** shows an annotated view of the Weight Management SmartForm.


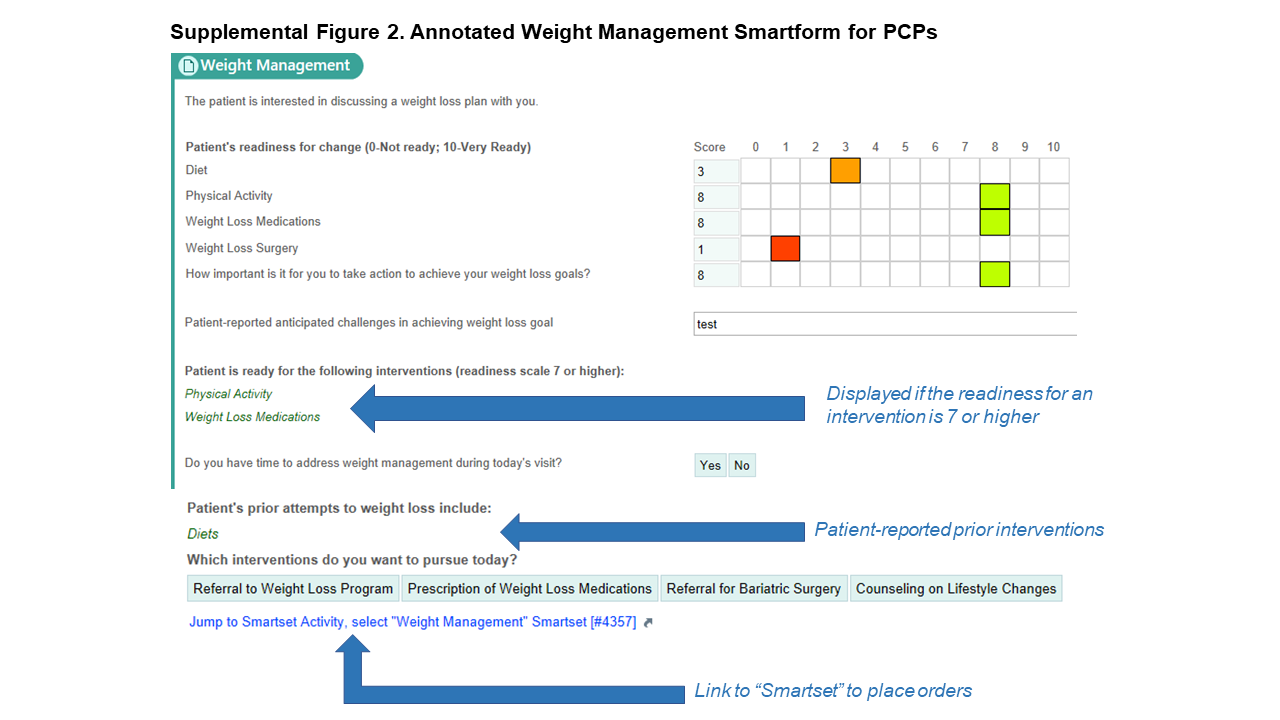


This SmartForm uses both text and color graphics to display information, which was identified as a key feature to ease use in the literature review. For example, green boxes indicate areas of readiness (i.e., score of 7 or greater on 10-point ruler per motivational interviewing practices) and these key areas are also identified in text under “Patient is ready for the following interventions” section – this design allows PCPs to quickly identify what treatment option may be best for this patient. At the end of the SmartForm, PCPs select what treatment option(s) they are recommending for the patient by clicking box(es) – “Referral to Weight Loss Program,” “Prescription Weight Loss Medications,” “Referral for Bariatric Surgery,” or “Counseling on Lifestyle Change” – and place any orders by clicking on the “Weight Management SmartSet” link.

While the example case in Supplemental Figure 2 displays a patient who is ready to make changes, PCPs could encounter a patient who wants to discuss weight management but does not indicate readiness to change in any area. Discordance between desire to discuss weight management and readiness for change is referred to as ambivalence. In this situation, the SmartForm provided PCPs a tool to resolve the patient’s ambivalence to change by helping to guide the conversation in concordance with motivational interviewing principles (**Supplemental Figure 3**).


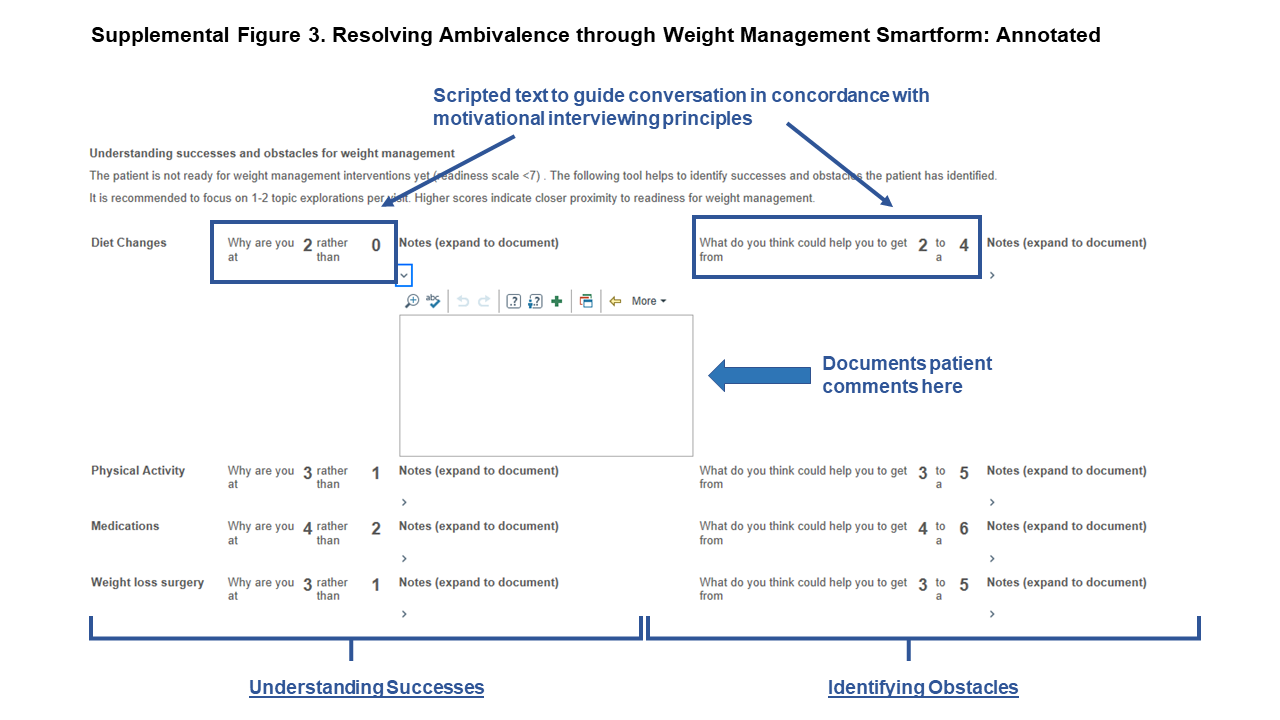


The primary care experts on the research team felt that it was important for PCPs to indicate on the SmartForm whether or not they had time to address weight management during the visit, as a scenario could occur where other health conditions need to take priority over weight management. Based on this feedback, PCPs could indicate on the SmartForm whether or not they had time to address weight management during the visit.
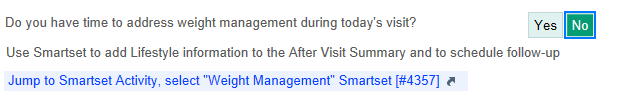
 If they did not have time, the PCP could select to opt out of discussing weight management, but were encouraged to order a follow-up visit for weight management and add patient education materials to checkout documents (“After Visit Summary”).

**Weight Management SmartSet.** The CDSS included a SmartSet, which is a curated list of orders. The Weight Management SmartSet was organized into 4 sections to mirror the 4 treatment options in the SmartForm – “Referral to Weight Management Program,” “Prescription of Weight Loss Medications,” “Referral for Bariatric Surgery,” and “Counseling on Lifestyle Change.” Under each of these headings, curated lists of relevant orders were included that were pre-populated to reduce PCP burden by allowing just a simple click (e.g., referrals, medications). Patient education materials were also included that the PCP could select with a simple click to be automatically included in the patient’s checkout materials (“After Visit Summary”). **Supplemental Figure 4** displays the curated list of orders for each treatment section.


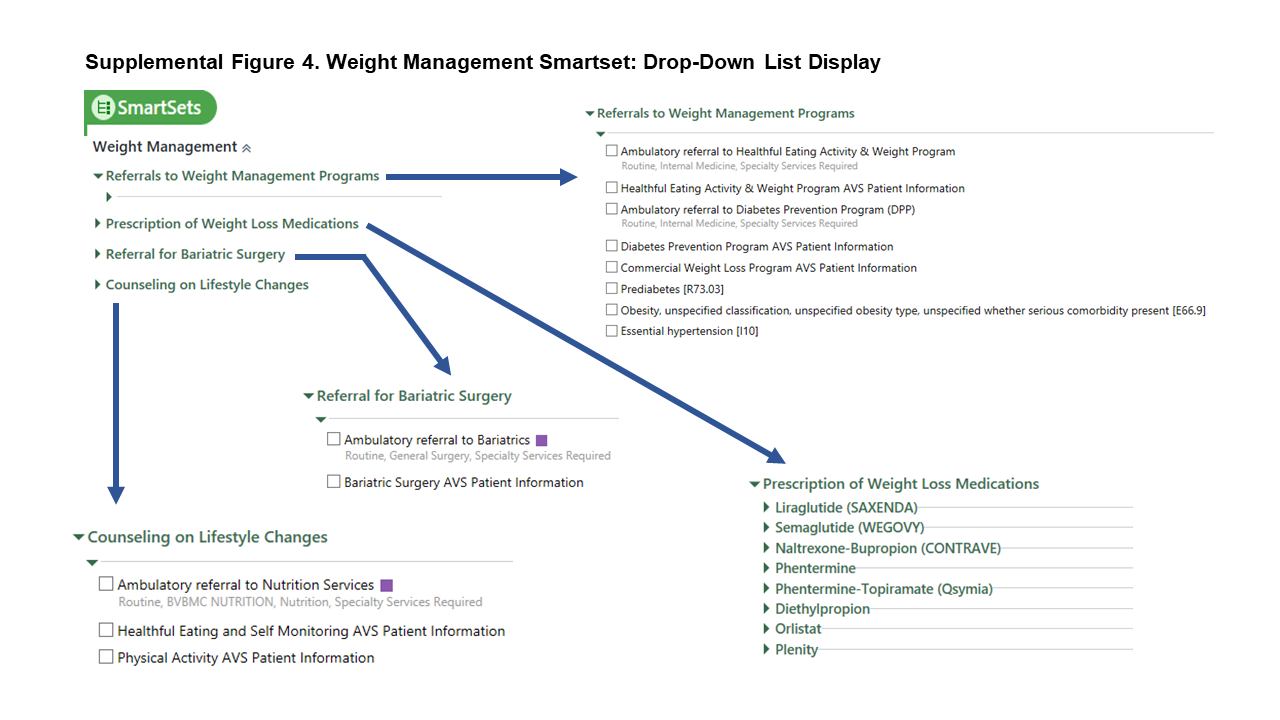


Of note, the “Prescription of Weight Loss Medications” section only included FDA-approved anti-obesity medications (or devices) available during the study period. Each medication had its down-drop down list that included pre-populated medication order(s) (including titration and maintenance dose, as applicable), pre-populated orders for any recommended monitoring (e.g., home blood pressure monitoring), patient education materials on the drug, and a follow-up weight management visit order.

**Automated Documentation.** The final component of the CDSS was the creation of “SmartLinks” to automatically populate information from the patient questionnaire and Weight Management SmartForm into PCPs’ visit notes. SmartLinks are phrases PCPs embed into their note templates that link to structured data in the EHR. During the visit, PCPs can add the SmartLink (if not already included in their note template) and update information by “refreshing” the SmartLink.

**
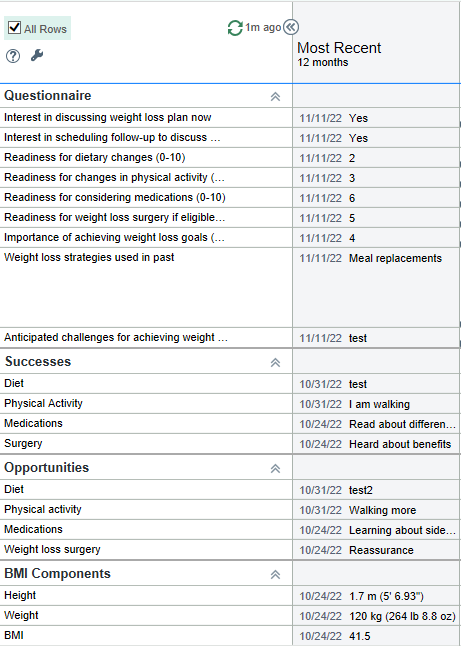
**

**Continued Use Over Time.** Patients who meet eligibility criteria would continue to receive the weight management questionnaire at upcoming visits to allow for tracking over time, which was hypothesized could be particularly helpful for PCPs working to resolve ambivalence. Prior patient responses could be viewed in the EHR through a specific section called “Synopsis” (displayed right). Of note, patients who indicated that they did not want to discuss weight loss and did not want a follow-up visit to discuss weight loss would not be eligible to receive the questionnaire again for 12 months.

**Supplemental Materials 2. Self-Efficacy Ratings at Baseline and 3 Months**


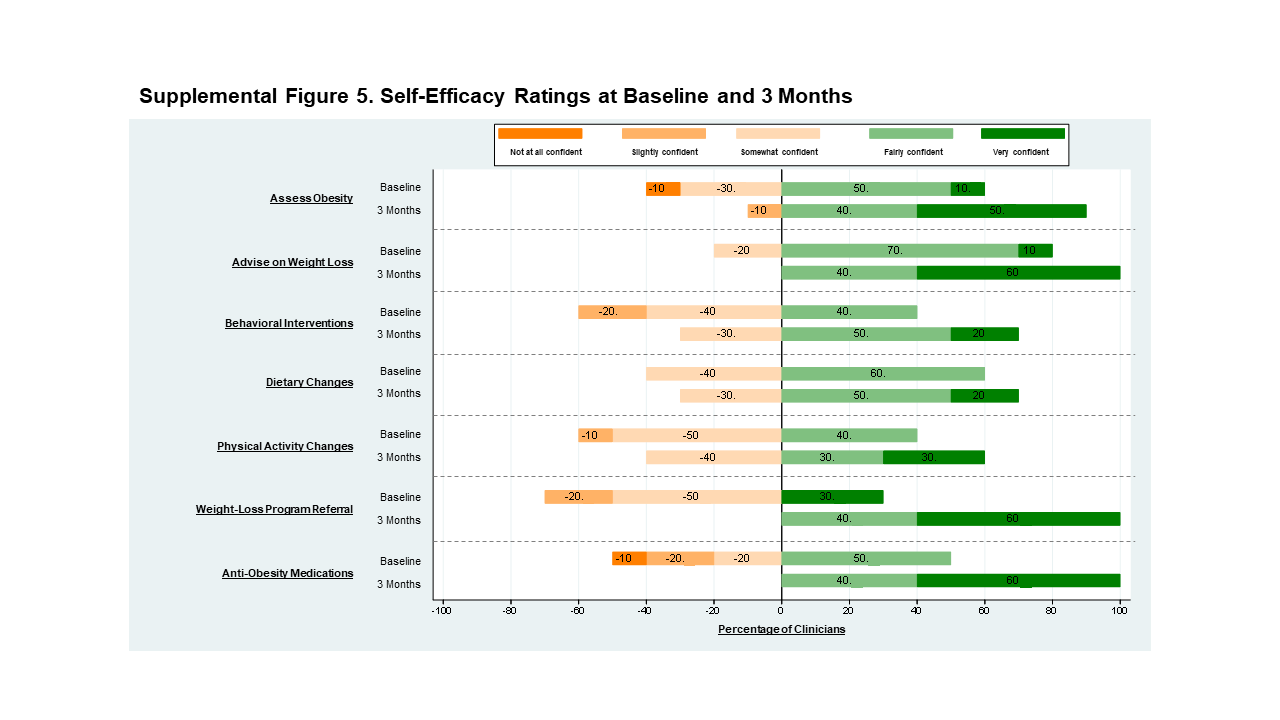


**Supplemental Materials 3. Frequency of Self-Reported Clinical Practice Habits at Baseline and 3 Months**


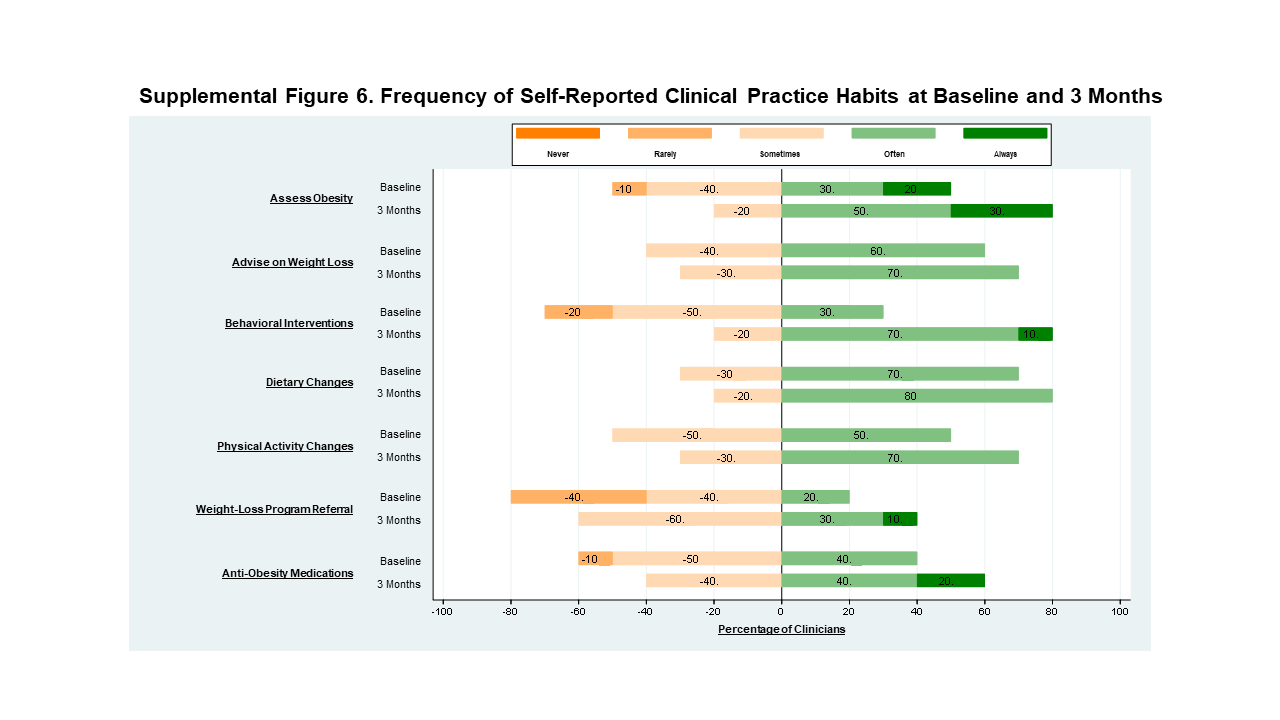


**Supplemental Materials 4. Overall Mean Self-Efficacy and Self-Reported Clinical Practice Habits at Baseline and 3 Months, by Individual Primary Care Practitioner**


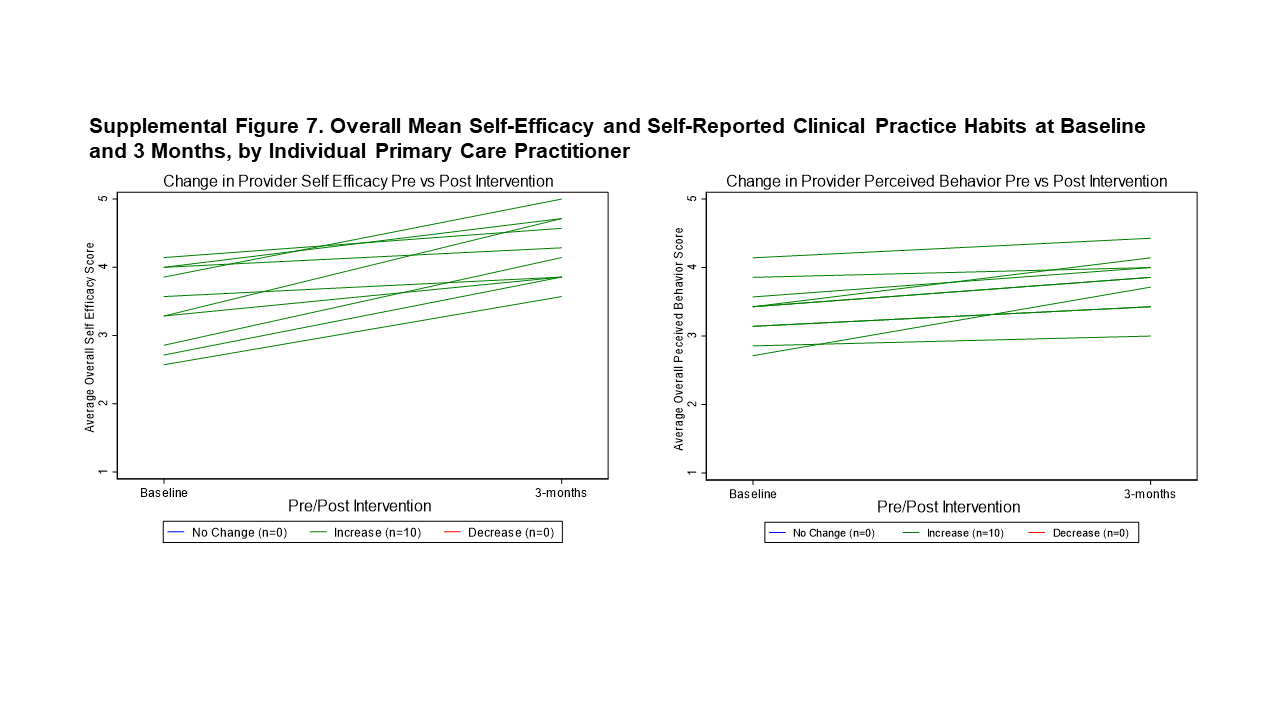


**Supplemental Materials 5. Chart Extraction Data: Results and Limitations**

**Results. Supplemental Table 1** displays the characteristics of the 381 extracted patient encounters along with the weight management elements documented in those encounters. Of note, 10 PCPs contributed records at the baseline and 2-week time points; 9 PCPs contributed records at 3 months (1 PCP was unable to contribute 3-month chart data due to unexpected medical leave).

| **Supplemental Table 1. Encounter Characteristics and Primary Care Practitioners’ Weight Management Documentation at All Time Points** | | | |
| --- | --- | --- | --- |
|  | **Baseline** | **2 Weeks** | **3 Months** |
| **# PCPs with available records** | 10 | 10 | 9 |
| **# Extracted encounters** | 128 | 138 | 115 |
| **Encounter Characteristics** |  |  |  |
| Patient age, % |  |  |  |
| 18-25 years | 0.8% | 0 | 0.9% |
| 26-40 years | 14.8% | 10.9% | 13.0% |
| 41-64 years | 47.7% | 53.6% | 45.2% |
| ≥65 years | 36.7% | 35.5% | 40.9% |
| Female patients, % | 67.2% | 72.5% | 70.4% |
| Patient race, % |  |  |  |
| White | 54.7% | 52.9% | 51.3% |
| Black | 32.8% | 40.6% | 40.9% |
| Other/unknown | 12.5% | 6.5% | 7.8% |
| Patient BMI in kg/m^2^, mean (SD) | 38.5 (7.4) | 36.4 (6.2) | 37.1 (6.9) |
| **Weight Management Documentation** |  |  |  |
| Obesity diagnosis in note, % | 40.6% | 43.5% | 41.7% |
| Components of weight management plan, % |  |  |  |
| Behavioral intervention | 6.3% | 6.5% | 8.7% |
| Dietary modification | 40.6% | 18.1% | 30.4% |
| Physical activity | 28.9% | 18.1% | 26.1% |
| Anti-obesity medication | 8.6% | 12.3% | 12.2% |
| Counseling approach, % | 0 | 1.4% | 0 |
| Weight-related diagnosis code billed, % | 43.0% | 37.7% | 40.0% |
| *Abbreviations: BMI – body mass index; PCP – primary care practitioner.* | | | |

Documentation of discussion of anti-obesity medications in the note increased over time from 8.6% at baseline to 12.2% at the 3-month mark. Behavioral interventions also increased from 6.3% at baseline to 8.7% at 3 months. Of note, PCPs continued to rely upon the prior SmartLink within the EHR to document obesity treatment plans (40.6% at baseline; 39.0% at 2 weeks; 30.0% at 3 months).

**Limitations.** Chart extraction was used to capture data and the frequency of weight management elements was reported, specifically obesity diagnosis, weight management plan, counselling approach, and weight-related diagnostic code. Documentation practices varied between PCPs – some provided succinct and detailed information about the treatment plan, while others provided scant information or documented a long list of information where it was difficult to ascertain the current treatment priorities. The clinical practice network and EHR did not require PCPs to document in a specific way. Some PCPs used medical scribes to write some of their notes and interface with the EHR, while others had trainees occasionally perform these activities – this introduced variability in documentation practices within each PCP. The variable documentation practices within the primary care visit notes limit the confidence in the quality of this data.

During the study period, the administration for the clinical practice network encouraged PCPs to use an existing SmartLink text within their notes to document an obesity treatment plan for all adult patients with BMI ≥30 kg/m^2^, which the administration would be tracking as part of a quality assessment. This existing SmartLink defaults to state that the plan discussed “included (but not limited to) diet, exercise and lifestyle modifications,” and PCPs must actively check other boxes displayed that state more specific strategies (e.g., daily calorie goal or prescription medication). During chart extraction, PCPs continued to use the existing SmartLink to document the obesity treatment plan. This behavior may have been driven by awareness that the clinical practice administration was monitoring their use of this specific SmartLink.

While weight-related history and prior/current weight management approaches were extracted, there were substantial differences in documentation practices related to patient history between PCPs (e.g., carrying forward of information from prior notes, lack of history section). These documentation practices made it challenging to determine weight history information obtained at that visit. Therefore, these outcomes were not reported.

**Lessons for Future Research and Implementation.** Future studies should consider tracking structured field EHR outcomes, such as referrals placed or medications prescribed, to better estimate treatments provided. The research team would recommend avoiding a chart extraction approach given the aforementioned data quality concerns. Implementation research using CDSS should also consider how to engage scribes and trainees, if they commonly work in the practice settings targeted. Finally, future research or real-world implementation of the CDSS should ideally incentivize the CDSS SmartLink as a quality metric equivalent to the existing one, which may support better quality PCP documentation of the obesity care plan. As patients are now able to access and read their visit notes (“open notes”), clear plan documentation in the note is increasingly important.
